# Effect of Plant Fiber on Early Properties of Geopolymer

**DOI:** 10.3390/molecules28124710

**Published:** 2023-06-12

**Authors:** Chun Lv, Dan Wu, Guoliang Guo, Yanming Zhang, Shuang Liu, Enxiang Qu, Jie Liu

**Affiliations:** 1 College of Architecture and Civil Engineering, Qiqihar University, Qiqihar 161006, China; 03269@qqhru.edu.cn (D.W.); 02559@qqhru.edu.cn (G.G.); 03421@qqhru.edu.cn (Y.Z.); 03508@qqhru.edu.cn (S.L.); 03318@qqhru.edu.cn (E.Q.); 2 College of Light-Industry and Textile Engineering, Qiqihar University, Qiqihar 161006, China; 3 Engineering Research Center for Hemp and Product in Cold Region of Ministry of Education, Qiqihar 161006, China

**Keywords:** geopolymer, plant fiber, early property, workability

## Abstract

Geopolymer (GP) is environmentally friendly, has good mechanical properties and long-term workability, and has broad application prospects. However, due to the poor tensile strength and toughness of GPs, they are sensitive to microcracks, which limits their application in engineering. Fiber can be added to GPs to limit the growth of cracks and enhance the toughness of the GP. Plant fiber (PF) is cheap, easy to obtain, and abundant in source, which can be added to GP to improve the properties of composites. This paper reviews recent studies on the early properties of plant fiber-reinforced geopolymers (PFRGs). In this manuscript, the properties of PFs commonly used for GP reinforcements are summarized. The early properties of PFRGs were reviewed, including the rheological properties of fresh GPs, the early strength of PFRGs, and the early shrinkage and deformation properties of PFRGs. At the same time, the action mechanism and influencing factors of PFRGs are also introduced. Based on the comprehensive analysis of the early properties of PFRGs, the adverse effects of PFs on the early properties of GPs and the solutions were summarized.

## 1. Introduction

At present, Geopolymers (GPs) are considered an environmentally friendly alternative to traditional building materials and an ecological material to replace traditional cement in recent years [1,2,3]. Compared with Portland cement, GP has less impact on the environment and can improve the sustainability of the structure, so it is an emerging alternative to ordinary Portland cement [4]. GPs have many advantages, such as low cost, environmental friendliness, and relatively low energy consumption. At the same time, it has high early strength, fast hardening, good compressive strength, and durability, and broad application prospects. The production process of GP is relatively simple, which has outstanding advantages in energy saving and carbon emission reduction [5]. In contrast to organic polymer materials, GPs have the advantages of high hardness, high strength, good thermal stability, and strong oxidation resistance. GPs have good mechanical properties, including compressive strength, corrosion resistance, and high-temperature resistance. Therefore, they have been used in many fields [6].

In general, the synthesis of GPs consists of an active solid silicaluminate precursor and activator solution. The source of precursor material is very rich. For example, kaolin, silica fume, fly ash, slag, and other industrial wastes [7]. The most commonly used are kaolin, fly ash, and slag. The activator solution acts as a binder, alkali activator, and dispersant. This method is relatively mature and widely used for the synthesis of alkaline solutions of activators such as NaOH and KOH with silicon and aluminum raw materials [8].

As mentioned earlier, GPs are an emerging sustainable material that meets the goals of energy conservation and environmental protection and can reduce carbon dioxide emissions in the environment. GPs are inorganic polymer materials with a three-dimensional network structure composed of Si-O_4_ and Al-O_4_ tetrahedral units. GPs are an environmentally friendly cementing material with low energy consumption and less pollutant emission in the production process [9,10]. However, similar to other traditional inorganic cementing materials, the brittleness of GPs limits their use in many fields. To avoid this phenomenon, fibers can be added to the GP matrix as reinforcements. The addition of fiber changes the GP matrix from brittle to ductile. In addition, it also increases the energy absorbed by the composite before the damage, including reducing the generation of matrix cracks [11,12]. Fiber is applied to the GP matrix not only because of its excellent recombination properties but also because of its simple application in the manufacturing process [13]. Because staple fibers are easily dispersed in the matrix, using staple fibers is an effective way to strengthen the GP matrix. The addition of fibers to GP can limit the growth of cracks and enhance the ductility, toughness, and tensile strength of composites [14,15].

Plant fiber (PF) has the advantages of low density and a high length-diameter ratio. It is not only cheap and readily available, but also comes from abundant sources and can be repeatedly processed and biodegradable [16]. At the same time, PF has high strength and low hardness, which can improve the brittleness of the matrix and the strength of the material when added to the composite. Therefore, PFs can be used as reinforcing materials to be added to GPs [17].

In recent years, PFs have been gradually applied to the development of engineering materials to improve the brittleness and other properties of cementing materials [18,19,20]. Among PFs, bast fibers are widely used as matrix-reinforcing materials due to their good properties and easy processing [21,22]. Othuman et al. [23] studied the effect of the addition of various bast fibers on the composite. These fibers include kenaf, ramie, flax, and jute. The content of fiber weight fraction is 0.45%. The results show that ramie fiber leads to the decrease of a slump of composites in workability. In terms of porosity and water absorption, adding jute fiber has the best effect. Abbas et al. [24] studied the influence of fiber content and length on the strength of cementing composites by adding kenaf fiber into the matrix. Petrella et al. [25] studied the performance of composite mortar with straw fiber as an admixture. It is found that the workability of straw composite mortar decreases with the increase in straw content. It is found that the mechanical properties of the straw composite are positively correlated with fiber length and inversely correlated with fiber dosage. It is found that there are more studies on the mechanical properties and long-term properties of plant fiber-reinforced matrix, but relatively few studies on the effect of PF on the early properties of the matrix. Ahmad et al. [26] studied the influence of fibers on the early properties of the matrix and found that coir fibers can significantly improve the cracking resistance of concrete, while synthetic fibers have adverse effects on the fluidity of slurry.

Research in recent decades shows that PF is feasible to replace synthetic fiber in GP composites. The research on the early properties of plant fiber-reinforced geopolymers (PFRGs) in recent years is reviewed. In this paper, the characteristics of PFs commonly used in GPs and the properties of fiber-reinforced geopolymers are briefly reviewed. The research on the early properties of PFRGs is discussed, including the rheological properties of freshly mixed slurry, early strength of GPs, early shrinkage deformation and cracking of GPs, etc. The action mechanism and influencing factors of early properties of GPs were introduced. The microstructure of GP in the early reaction process was analyzed and discussed.

## 2. PFs and Their Characteristics

PFs are abundant, widely distributed, and have many other advantages. They are not only low-cost but also have good mechanical properties and are biodegradable [27,28]. The three main components of PF, cellulose, hemicellulose, and lignin, constitute the supporting skeleton in plants and exist in the form of the cellulose-hemicellulose-lignin bond [29,30,31]. Cellulose consists of microfibers that form the reticular skeleton of plant cell walls. Hemicellulose and lignin, as binders and fillers, are distributed among the microfibers. The different types of PFs commonly used for matrix reinforcement are shown in Table 1.

As can be seen in Table 1, PFs have low density and acceptable mechanical properties and are becoming more widely used. However, these PFs have some disadvantages, including poor bonding to the matrix, low durability, and reduced workability of fresh composites with high fiber content [67,68,69].

**Figure 1:**
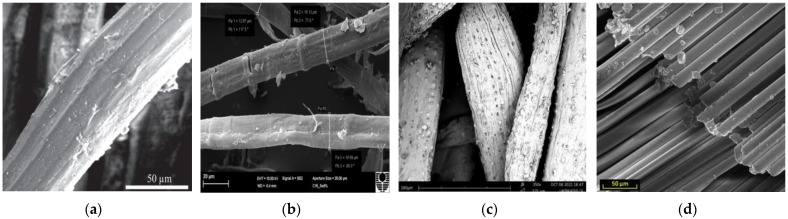
Scanning electron microscopy of surface morphology of fibers; (**a**) Changes in surface structure of jute [42]; (**b**) Changes in flax diameter [71]; (**c**) Changes in surface structure of coir [20] and (**d**) Surface structure of basalt [70].

However, compared with other types of fibers, PFs have unique properties that enable them to bond effectively to the substrate. Figure 1a–d [20,42,70,71] show the surface morphology of jute fibers, flax fibers, coir fibers, and basalt fibers used in relevant studies. The figure shows the rough surface of PFs and the changes in the surface structure of the fibers. In contrast to these PFs, basalt fibers, as traditional inorganic fibers, have a smooth surface and no change in diameter. See Figure 1d. These fibers with rough surfaces act as the GP reinforcement body so that the interface bond adhesion between the GP matrix and the reinforcement fiber will increase, which will lead to higher strength of the composite.

## 3. Plant Fiber-Reinforced Geopolymers (PFRGs)

**Figure 2:**
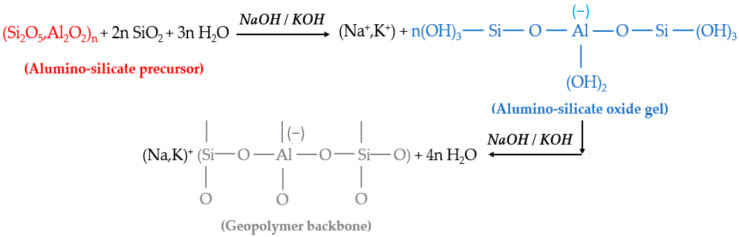
The chemical structure of the GPs.

Cement produces gels due to the hydration of mineral particles, but the reaction systems of GPs and cement-based materials are quite different. Cellulose is the most important component of PFs, and its chemical formula is (C_6_H_10_O_5_)_n_. The chemical structure of the GPs is shown in Figure 2 [18]. PFs are very prone to alkaline degradation in the cement-based material matrix, while the degradation degree of PFs in the GP matrix is much lower [72]. The composite of PFs and GPs can overcome the disadvantage of poor durability caused by fiber degradation of composite materials, and its performance is better than that of PF-reinforced cement-based composite materials in some respects [73,74]. GPs not only have the advantages of low cost, environmental friendliness, and relatively low energy consumption, but they also have good compressive strength, durability, and heat resistance, as well as high fire and heat resistance [75].

Some researchers believe that the reaction process of GP mainly consists of dissolution, diffusion, polymerization, and solidification. First, when aluminum silicate raw materials are dissolved in an alkali activator solution, many silicon and aluminum monomers can be produced. The silicon and aluminum monomers gradually diffused from the surface to the inside and then condensed rapidly to form the oligomer gel phase of silicon and aluminum. Finally, the silica-alumina oligomer gel phase solidifies and hardens into GP concrete [76,77]. However, GP composites are also similar to cement-based materials in that they have relatively low tensile and bending strength, which limits their application in many fields. The addition of fiber decreases the brittleness and increases the toughness of the GP matrix, enabling it to achieve a wide range of engineering applications [78,79]. Considering environmental concerns and material energy-saving requirements, the use of PFs such as jute, hemp, bagasse, and sisal is considered to be an effective alternative to synthetic fibers in reinforced composites [80].

### 3.1. Compatibility and Water Absorption of PFRGs

When PF is added to the GP matrix, a series of changes will occur in fiber properties. The compatibility between PFs and GP matrix will affect the workabilities and mechanical properties of composites to a great extent. Tan et al. [81] studied the compatibility of PFs and GPs by evaluating the effect of PFs on the change in polymerization temperature of the GP matrix. The results showed that the maximum temperature of fiber-reinforced geopolymerization was lower than that of pure geopolymerization, and the maximum temperature of geopolymerization was delayed than that of pure geopolymerization, indicating that the geopolymerization was inhibited by adding PFs. In addition, due to the inhibition effect of PFs, the geopolymerization curve of PFRG drops more gently than that of pure GP. Camargo et al. [82] also used the evaluation method of PF-reinforced cement-based materials to analyze the compatibility between PF and GP slurry. Alomayri et al. [83] prepared cotton fabric-reinforced GP composites through layup technology. By adjusting the compatibility between cotton fiber and GP matrix, the interfacial bonding property is improved, and the bending property and fracture property of the composites are improved effectively. Fonseca et al. [84] used hot water treatment, keratinization, and alkaline hybridization pretreatment, respectively, to analyze the effects of pretreatment on the characteristics of pine fiber, soil palm fiber, shavehead fiber, and jute fiber. The results showed that the best compatibility between PFs and GP matrix was obtained by the modified pretreatment methods of hybridization and keratinization. Using NaOH alkaline treatment and hybridization, the palm cellulose chain can be reorganized, the fiber can be divided into more fibrils, and the ductility and mechanical properties of the palm fiber can be improved greatly.

The PF itself has an internal cavity structure [29,30,31]. Based on this property, the addition of PF has a great influence on the absorbency and density of the composite. Generally speaking, the water absorption of the material is related to the connection mode of the inner pore of the composite. Zhou et al. [58] found that the water absorption of FRGP was significantly improved when cotton stalk fibers were embedded into the matrix. Increasing the amount of fiber by 0.5% increased its water absorption by about 28%. It was also found that as the fiber aspect ratio increased, the cotton fiber would gradually float on top of the fresh composite, resulting in higher water absorption.

Fiber can affect the density of composites as well as the water absorption. Liu et al. [85] studied wheat straw GP biological insulation material. The test results show that the water absorption rate of the composite is 32 to 107%, the moisture absorption rate is 4 to 33%, and the density is 235 to 894.1 kg m^−3^. Pimraksa et al. [86] believed that GP density could be used to characterize the degree of polymerization of GPs to a certain extent. The higher the content of cotton stem fiber, the lower the GP density. Belhadj et al. [87] studied the relationship among the density, water absorption and porosity of wheat stem reinforced concrete. It was found that with the increase in stalk content, wheat density decreased and water absorption increased. Wongsa et al. [88] added sisal fibers, coir fibers, and glass fibers to GPs at volume fractions of 0, 0.50, 0.75, and 1.00%. The mixtures are indicated by fiber content and fiber type, as shown in Table 2. It can be seen that fiber content and fiber type are related to water absorption. The water absorption rate of sisal and coir is much higher than that of glass, which is related to the characteristics of PF. The water absorption rate of GP with glass is lower than that of GP without fiber.

### 3.2. The Interface between PFs and GP Matrix

The interface characteristics and adhesion of fibers and matrix are key factors in the interface control technology of composites [52,80]. The bond between the GP matrix and the fiber is similar to that of the cement matrix. Under the action of external tension, composites fail mainly through fiber fracture and pull-out, and fiber pull-out is the main factor leading to composite failure [89,90]. Therefore, the mechanical properties of composites depend on the bonding force between fiber and matrix [91]. Wei et al. [92] studied the influence of binary blends of kaolin and nanoclay on the interfacial bond strength of sisal fiber in the cement matrix. The addition of kaolin and nanoclay only slightly increased the compressive strength of mortar, but significantly increased the interfacial bond strength between fiber and cement matrix. The interfacial bond strength of single cement is 0.321 MPa, and the bond strength between sisal fiber and the matrix is 0.743 MPa by adding 30% kaolin and 2% nanoclay, which increases 131.46%. However, as the amount of kaolin and nanoclay continues to increase, the cement is diluted and the compressive strength decreases.

In fact, by adding kaolin and nanoclay, the porosity and pore size distribution of cement mortar is reduced [92,93]. On the other hand, the silicates and aluminates produced by kaolin and nanoclay react with the calcium hydroxide produced by the hydration of cement, resulting in more C-S-H gels, which promote the interface adhesion between fiber and matrix.

## 4. Workability of PFRGs

GP is considered a green alternative to Portland cement. Previous studies on GPs have focused on their chemical and mechanical properties, microstructure, and potential applications, but few researchers have focused on their rheological behavior. In the fresh state, good workability of PFRGs is a prerequisite for better mechanical properties [94,95]. The workability of cement concrete is usually evaluated by the workability index. Similar to the workability evaluation of cement concrete, the workability of GPs can also be measured by slump test, fluidity test, and the relationship between flow time, flow diameter, and yield stress [96,97]. The early properties of GPs are different according to the different types and compositions of silicon and aluminum raw materials, and the types and proportions of alkali activators. There are fundamental differences between ordinary Portland cement and GP in flow characteristics [98,99]. Favier et al. [4] found that, unlike ordinary Portland cement, the colloidal interaction between metakaolin particles of GP was very small, and its viscosity was mainly controlled by the high viscosity of the suspended alkaline silicate solution. Therefore, the current technology used to improve the rheological behavior of cement is not very effective for GPs.

### 4.1. Effect of Conventional Fibers on the Workability of GPs

Slump value is one of the important characteristics of the workability of fresh concrete. Slump tests can judge the workability and the freshness of the mixture. Compared with other fibers, steel fiber, and other conventional fibers are considered to be one the potential reinforcement fibers in GP composites. These fibers can enhance the flexural strength, tensile strength, ductility, and toughness of GP composites [70,100]. However, steel fibers have a negative effect on the porosity and workability of GPs [101]. It is found that the slump value of GP is 72% higher than that of cement concrete due to its viscous flow characteristics. However, the addition of steel fibers will hinder the flow of GP slurry and thus reduce its workability [102].

**Figure 3:**
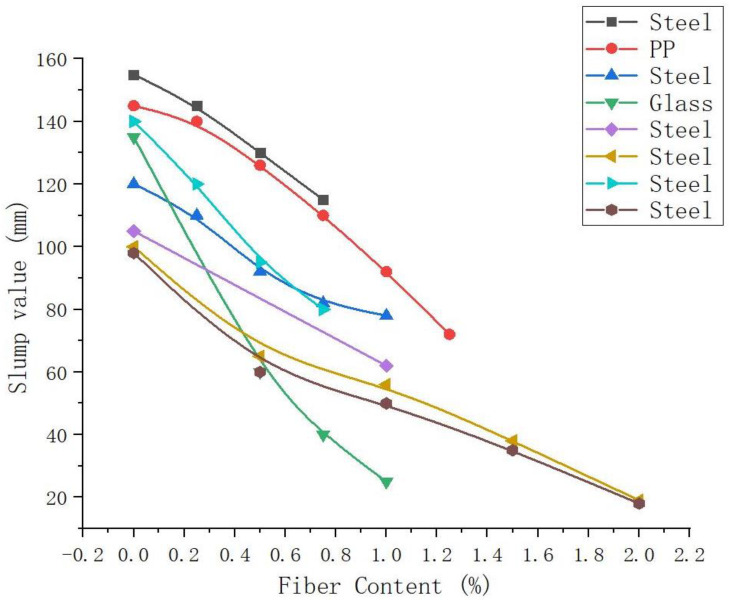
Effect of traditional fiber content on slump of GP slurry [88,101,102,103,104,105,106].

Figure 3 shows the relationship between the content in steel fiber and the slump value of GP slurry. It can be seen that with the increase in fiber content, the slump of GP slurry continues to decrease. Generally speaking, the addition of fibers can improve the compressive strength, splitting tensile strength, and bending strength of the matrix. However, the improvement was more significant in the splitting tensile strength and bending strength. It can be seen from Figure 3 that the slump height of the GP mixture with 0.25 vol% fiber content is greater than 90 mm. For mixtures with 0.5 to 1 vol% fiber contents, most slump heights are between 50 and 90 mm. When the fiber content exceeds 1 vol%, the slump value of the mixture is less than 50 mm. Therefore, to maintain adequate fiber dispersion, a fiber volume fraction of about 0.75% is recommended. PP fiber is similar to steel fiber, and glass fiber declines faster. This is related to the length of the fiber used in the test. At the same time, the glass fiber is not easy to be evenly dispersed in the matrix and easily wound into clusters, which affects the workability of the fresh mixture.

Bashar et al. [107] conducted an experimental study on palm oil fly ash GP with steel fiber as reinforcement and oil palm shell as crude aggregate. The influence of two aspect ratios and three volume fractions (0.25%, 0.50%, and 0.75%) steel fibers on the tensile and fracture properties of the GP was investigated. The toughness and equivalent bending strength ratio of the reinforced GP are higher than those of the non-reinforced GP. In addition, the high content of palm oil fly ash water absorption results in reduced workability. In addition, the lightweight of palm oil fly ash causes the slump of the paste to be close to zero. However, with proper vibration, no honeycomb-pockmarked surface appeared during demolding. It shows that the GP slurry can use the least water under the stable material amount, thus achieving zero slump.

### 4.2. Effect of PFs on the Workability of GP

In contrast to traditional inorganic fibers, the sugar in PF is easy to hydrolyze under the action of alkalinity in the matrix. Hydrolysate plays an anticoagulant role in the hardening of the composite. At the same time, the fluidity of the slurry will be significantly reduced, and the slump loss of the slurry will be accelerated.

Wongsa et al. [88] found that the workability of sisal and coir fiber-reinforced GP slurry was lower than that of common GP slurry. Sisal fibers have rough surfaces, irregular stripes, and porous textures. Sisal fiber reduced the workability of the GP mixture the most compared with coir fiber. In addition, with the increase in PF content, the fluidity of the slurry further decreased. Silva et al. [42] studied the effect of jute and sisal fibers on the workability of GPs. The results show that the workability of GP composites reinforced by sisal fiber decreases less than that of jute fiber under the same fiber contents. When the fiber content was 2 wt.%, the slump value of GP slurry containing sisal was 50% higher than that of slurry containing jute. This is due to the low aspect ratio of sisal fiber. This is consistent with relevant literature [108,109], i.e., the workability of fiber-reinforced cementitious materials decreases with the increase of aspect ratio.

**Figure 4:**
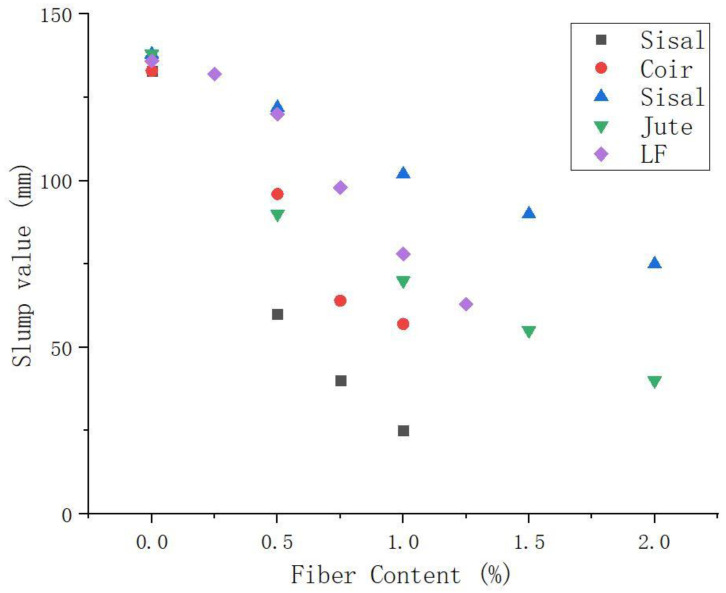
Effect of PF content on the slump of GP [42,88,106].

Abbas et al. [45] studied the influence of kenaf fiber length and content on the workability of the matrix through slump height. The increase in fiber volume fraction significantly reduces the processing capacity. The fiber volume fraction of length 20 mm increased from 0.75% to 1.5%, and the slump height was reduced from 50 mm to 37 mm. Similarly, mixtures prepared with 30 mm and 40 mm kenaf fiber showed a loss in machinability as the fiber volume increased from 0.75% to 1.5%. Haddaji et al. [110] reported the same pattern of reduced workability when sisal, coir, and lignin fibers were added to GP mixtures. At constant volume fraction, the shorter length of 20 mm fiber has less effect on workability. Comak et al. [111] reported the same behavioral trend using 6–18 mm hemp fibers. This is consistent with the performance of Nayak et al. [112] for fresh mortars reinforced with jute, sisal, and polypropylene fibers of 1% to 2% content. The effect of PFs on the fluidity of GP slurry is shown in Figure 4. The increase in PF contents results in a decrease in fluidity, as shown in Figure 4. Mixtures containing PFs showed the greatest reduction in workability compared to mixtures containing steel fibers or other conventional fibers. There is a relationship between the fluidity of composites and the amount of PFs. The degree of workability loss of fiber-reinforced GPs is dependent on the fiber type, aspect ratio, and content in the mixture. Due to the hydrophilicity of PFs, the workability reduction due to the addition of PF is higher than that of steel fibers and inorganic fibers in the same GP.

Similar to the above studies, Su et al. [106] studied the performance of lignin fiber-reinforced GPs with a weight content of 0.25 to 1.25%. It shows that GP mixtures with high fiber content have lower workability. In addition, Su et al. observed that the workability of PFRGs decreased the most compared to inorganic fiber-reinforced GPs.

In summary, the workability loss of different types of fiber-reinforced GP mixtures is affected by their type, aspect ratio, and content in the mixture. The workability reduction of PF is higher than that of steel fiber and inorganic fiber with the same GP matrix. This is due to the high water absorption of PFs. Therefore, pre-wetting or pretreatment of PFs before the addition of GP mixtures can reduce the absorbent chemical composition of the fibers.

From the perspective of low carbon and environmental protection, relevant personnel also carried out research on the related properties of waste PFRGs [113]. Liu et al. [85] studied the influence of four variables, including thermal properties, mechanical properties, hydraulic properties, and microscopic morphology, on the properties of biological thermal insulation materials using wheat straw as aggregate and GP as binder. Duan et al. [62] studied the fresh properties of fly ash GP with sodium silicate and sodium hydroxide solution mixed with 0 to 20% sawdust, including workability, setting time, drying shrinkage, mechanical strength, and microstructure. The results show that the workability of GP is affected by the addition of sawdust. The sawdust content is inversely proportional to the setting time.

Malkawi et al. [114] used palm oil clinker as a lightweight aggregate to prepare GP composites. Compared with the control group, the workability of light aggregate GP slurry decreased with the increase of palm oil clinker content. This is mainly due to the absorbability of palm oil clinker. In the mixing process, a large amount of alkaline solution is absorbed, and a drier fly ash paste is produced, which leads to the workability of the slurry being reduced. In addition, low-density palm oil clinker will produce a smaller slump in value. On the other hand, palm oil clinker has a rough and spiky surface, which will also help to reduce slump value. When palm oil clinker content increased by 25%, slump value decreased by nearly 21%. It was also found that the addition of oil palm trunk fiber had a significant effect on the workability of GPs. When 1% oil palm trunk fiber was added, the apparent processability of light aggregate GPs was reduced by 20%, and that of light aggregate GPs was reduced by 73% when 3% oil palm trunk fiber was added.

**Figure 5:**
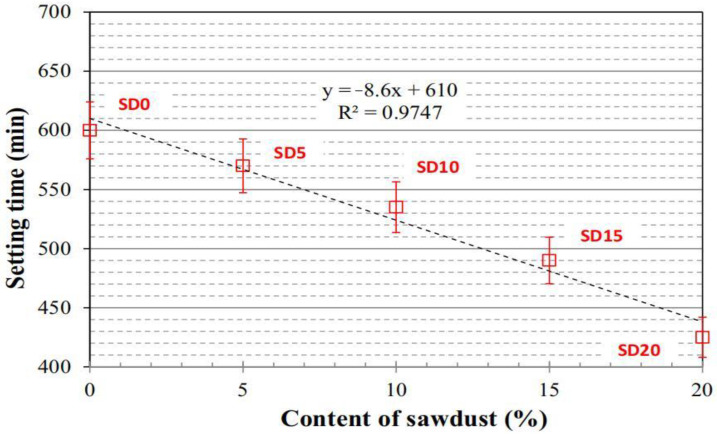
Relationship between sawdust fiber content and setting time of GPs [62].

In addition, the effect of setting time is also an important index to evaluate the rheological properties of GPs. Ferreira [115] et al. investigated the effects of microcrystalline cellulose on the mechanical and microstructure properties of Portland cement and GP slurry. The use of microcrystalline cellulose increased the compressive strength and stiffness of both studied binders at 7 days. After 28 days, the mechanical properties of GP decreased due to the degradation of microcrystalline cellulose. The addition of microcrystalline cellulose accelerated the setting time of GP by 15%, but delayed the setting time of the cement slurry. It is reported that the setting time of reference GP is about 3.8 h. With the addition of 2% microcrystalline cellulose, this time was reduced to 3.2 h, a reduction of 15%. In contrast, when microcrystalline cellulose was added to the cement slurry, the setting time increased by 41% from 2.3 h to 3.9 h. Given this phenomenon, Hoyos et al. [116] believed that the interaction between microcrystalline cellulose, hydration products, cement particles, and water generated a waterproof barrier to the anhydrous particles of cement and delayed the hydration reaction. On the other hand, the addition of microcrystalline cellulose to the GP accelerates the setting time. This may be due to heat generated during cellulose degradation, which enhances the polymerization reaction. This is also reflected in the relationship between sawdust fiber content and the setting time of GPs in Figure 5 [62].

### 4.3. The Factors Affecting the Workability of GP

Many factors affect the rheological properties of GPs. Due to the high viscosity of sodium silicate solution, the rheology of GPs is quite different from that of ordinary concrete [117]. When cement is hydrated, slump time loss will occur. On the one hand, there is less free water in the system, on the other hand, the effect of admixtures is weakened. In addition, a large number of hydration products such as Ca(OH)_2_ and C-S-H will be produced in the hydration process of cement, which will increase the viscosity of the system and thus increase the slump loss of concrete. Adding admixtures to concrete is beneficial to maintaining the good fluidity of concrete. The surface of mineral admixtures is smooth and dense. When mixing concrete, they are dispersed among cement particles, and these dense particles act as dispersants.

The higher ratio of sodium silicate to sodium hydroxide in the GPs exacerbates the viscosity effect and further reduces the slurry flow [118,119]. Similarly, due to the faster dissolution rate of silica and alumina, an increase in the concentration of the alkali activator tends to reduce the rheology, thus speeding up the GP reaction.

In addition, increasing the volume of the activator solution has been shown to improve rheology without significantly affecting the compressive strength of the mixture due to an increase in its liquid-solid ratio [120]. Su et al. [106] also came to the same conclusion. They studied the effect of the liquid-solid ratio on the setting time of GPs and found that when the liquid-solid ratio was 0.9, the initial setting time of GPs was 21 min, and the final setting time was 29 min. When the liquid-solid ratio is 1.3, the initial setting time of GPs was 60 min, and the final setting time was 82 min. The higher the liquid-solid ratio is, the longer the setting time is.

The addition of PFs will reduce the fluidity of the GP slurry and shorten the setting time of the slurry. The addition of some materials, such as rice husk ash, also affects the rheological properties of the GPs. Das et al. found that due to the absorption characteristics of rice husk ash particles, rice husk ash has a high water requirement, and insufficient water content will lead to faster hardening, thus reducing the processing capacity and setting time [121,122].

## 5. Early Strength of PFRGs

PFs affect the early strength of GPs, including compressive strength, flexural strength, and tensile strength. The results show that the PF can shorten the setting time of the matrix and improve the ductility and tensile strength of composites. Its influence on the composites is mainly manifested in the enhancement of toughness and the decrease of compressive strength [123,124].

### 5.1. Effect of PF on the Compressive Strength of GPs

The early strength of GPs is higher than that of cement concrete. Saranya et al. [108] found that under normal temperature curing conditions, the compressive strength of GPs reached 95% of the designed strength within 7 days, while the compressive strength of cement concrete only reached 50% of the designed strength within 7 days. An appropriate number of fibers distributed evenly in the matrix can enhance compactibility, reduce porosity and crack, and thus improve the compressive strength of the composite. Kavipriya et al. [125] found that when the common aggregate in the composite was replaced with 10%, 20%, and 30% bamboo aggregate, the strength of the GP matrix did not decrease, while the strength of cement concrete decreased more.

Both traditional inorganic fiber and PF can affect the compressive strength of the GP matrix. Korniejenko et al. [59] analyzed the effects of short coir, glass, and carbon fibers on the compressive strength of fly ash GP, which were 1, 2, and 5% by weight of fly ash. Samples from each series were tested for compressive strength after 7, 14, and 28 days. The results show that the compressive strength of the composites increased by 25.0% to 56.5%. Ayeni et al. [20] also studied GP composites with coir fiber content. It was found that the compressive strength increased from 16.91 N/mm^2^ to 21.25 N/mm^2^ when 0.5% coir fiber was added. Korniejenko et al. [47] analyzed the mechanical properties of GPs reinforced with different PFs, such as cotton, sisal, Lafite, and coir. The results show that the mechanical properties of composites can be improved by adding a proper proportion of PFs. At the same time, it is found that the compatibility between Lafite fiber and GP matrix is poor, and the interface between fiber and GP matrix lacks adhesion.

Excessive fiber addition and uneven distribution in the matrix have adverse effects on the compressive strength of GPs [126]. Lazorenko et al. [127] randomly reinforced GP composites with flax fibers containing 0.25 to 1.0 wt.% by weight and demonstrated that flax fibers caused a decrease in the compressive strength of GPs. Zhou et al. [58] found that the addition of cotton stalk fiber reduced the density and compressive strength of the GP. Fonseca et al. [84] found that the fiber content was inversely proportional to the compressive strength of the composite by mixing different qualities of pine fiber, palm fiber, shaven grass fiber, and jute fiber into the GP matrix.

Abbas et al. [45] studied the influence of kenaf fiber length and volume fraction on the compressive strength of GPs. The compressive strength of GP composite is greatly affected by the length and volume content of kenaf fiber, and the strength decreases with the increase of fiber length and volume fraction. After curing for 7 days, the compressive strength of the GPs without fiber was 52.9 MPa, while the strength of the GPs with 0.75% kenaf fiber decreased to 48.9 MPa. However, the fiber content increased from 0.75 to 1.5%, and the compressive strength of GPs decreased from 48.9 to 37.7 MPa. On day 56 and day 90, the strength of all samples showed a similar decreasing trend as the length and volume content of kenaf fibers increased. Wongsa et al. [88] also reported that the dispersion of coir and sisal fibers in the GP matrix was poor at 1% fiber addition. When the fiber content increased from 0.5 to 1.0%, the compressive strength of the two fiber-reinforced GPs decreased by 26.4% and 11.5%, respectively.

The effect of waste fibers such as straw on the compressive strength of the GP matrix is similar to that of sisal and coir fibers. Workiye et al. [128] prepared corn straw fiber-reinforced GPs with weight contents of 0, 0.1, 0.2, 0.6, and 1%, and conducted compressive strength tests. The measured compressive strength ranges from 16 to 27 MPa. It indicates that the appropriate addition of corn straw fiber can improve the compressive strength of GPs. Furtos et al. [129] synthesized a new wood fiber-reinforced GP composite at room temperature and cured it at 90 °C for 24 h. An amount of 5 wt.% sand was added for GP internal fixation and 5, 10, 15, 20, 25, 30, and 35% wood fibers in different proportions were added. The experimental results showed that the mechanical properties decreased with the increase in wood fiber content. Compressive strength decreased from 39.17 to 31.79 MPa. Research has shown that adding 15% wood fiber may be the limit of the PFRGs.

### 5.2. Effect of PF on the Flexural Strength of GPs

The mechanical properties of PFs can improve the flexural strength of GPs, which is due to the high tensile strength and elastic modulus of PFs. The tensile stress exerted on the composite matrix can be transferred to the fiber through the interfacial bonding between the fiber and the GP matrix. In GP composites, fiber types, and properties are more effective than matrix properties in improving fracture properties such as flexural strength. Fiber properties control the failure mechanism and fracture characteristics of composites.

Lazorenko et al. [127] showed that the flexural strength of GPs increased from 4.0 to 4.9 MPa when 1.0% flax fiber was added to GPs. In contrast to the brittle GP matrix, the composite exhibits plastic failure characteristics and has a high residual bearing capacity, which can keep the specimen intact even after being subjected to a large mechanical influence. The test results of wood fiber-reinforced GP also showed that the flexural strength of GP increased from 7 to 10.76 MPa with the increase in wood fiber content [129].

The flexural behavior of PFRGs is similar to the compressive behavior. Zhou et al. [58] studied cotton fiber-reinforced GP and found that the flexural strength of GP increased slightly with the addition of fibers. The flexural strength of the 1% cotton fiber sample is the highest, i.e., 3.52 MPa, which is 10% higher than that of the sample without cotton fiber. Due to the fracture and energy consumption of fibers, the toughness is improved, while the fracture morphology of non-fiber composites is relatively smooth, indicating obvious brittle damage characteristics. Alomayri et al. [57] also analyzed the flexural strength of cotton fiber-reinforced GPs and found that the flexural strength increased from 10.4 to 11.7 MPa when 0.5% cotton fiber was added to the GPs. The flexural strength and fracture toughness were improved. However, adding more cotton fibers (0.7, and 1.0 wt.%) resulted in a decrease in flexural strength. Wongsa et al. [88] added 0.5, 0.75, and 1.0% sisal fiber and coir fiber into GPs, which significantly improved the tensile and flexural strength properties. The results show that there is a positive correlation between fiber content and flexural strength within a certain range. However, the strength of fiber decreases obviously when the content of fiber exceeds a certain value.

Abbas et al. [45] investigated the influence of kenaf fiber with a length of 20 to 40 mm and volume fraction of 0.75 to 1.5% on the engineering performance of GPs through the experimental test of kenaf fiber-reinforced GPs. After kenaf fiber was added, the splitting tensile strength and flexural strength of GPs increased, but the compressive strength was not increased significantly. The splitting tensile strength and flexural strength of kenaf fiber-reinforced GPs with a length of 30 mm and fiber volume fraction of 1.25% increased by 20% and 27%, respectively, compared with common GPs at 28 days of curing.

Korniejenko et al. [59] found that the flexural strength of composites with carbon fiber content of 1% and 2% increased significantly, by 62.4% and 115.6%, respectively. When 1% glass fiber and 2% coir fibers were added into the matrix, the flexural strength of the matrix increased slightly, to 4.5% and 5.4%, respectively. For the addition of 2% glass fiber, the flexural strength value did not change compared to that of pure GPs. However, the flexural strength values even deteriorated for GPs with other fiber content, such as 5% glass fiber and 1% and 5% coir fiber.

Similar to flax fiber, cotton fiber, sisal fiber, and coir fiber, related studies found that sawdust produced consistent results in the GP matrix [62]. Sawdust had little effect on compressive strength before curing for 14 days but had a positive effect on compressive strength after curing for 28 days. The addition of 5% sawdust has little effect on flexural strength and has nothing to do with curing days. The flexural strength increases with the increase in sawdust content. With the increase in sawdust content, the porosity decreased and the matrix was compact. The addition of wood chips results in the formation of optimal microstructure. The mechanical properties are in good agreement with the microstructure. Table 3 shows the experimental data of early flexural performance of GP composites with different PF content.

As can be seen from Table 2, the content of PF as reinforcement fiber of the GP matrix can range from 0.25 to 5%, usually around 1%. The content of lightweight aggregate as the matrix of GPs can reach more than 20%. The early (7-day and 14-day) flexural strength of PFRGs can reach 80% of the 28-day flexural strength. The early flexural strength of the composite can be improved effectively by adding PF into the GPs. Studies have shown that compared with the compressive strength, the fiber type and content have a significant effect on the improvement of the flexural strength and splitting tensile strength of the GP [88]. Table 4 shows the effects of different types and volume contents of fibers on the flexural to compressive strengths and splitting tensile to compressive strengths ratios of GPs.

### 5.3. Microscopic Analysis of the Early Reaction of GPs

Through microscopic analysis of the early reaction process of GPs, researchers have further understood the structure and formation mechanism of the early reaction of PFRGs, to better understand their early properties. With the further study and development of the chemical reaction and mechanism of GPs, other early characterization techniques have been gradually developed and applied.

**Figure 6:**
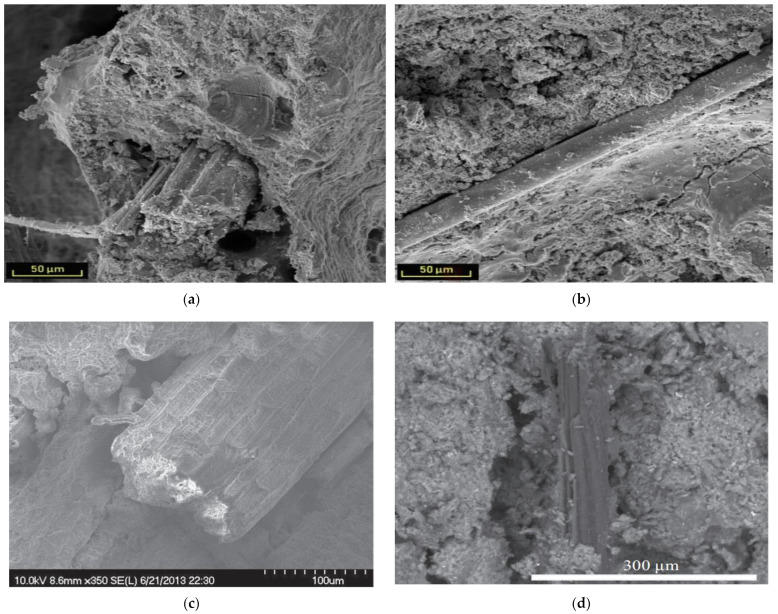
SEM micrographs of fiber-reinforced GPs: (**a**) Flax fiber fracture; (**b**) Basalt fiber [70]; (**c**) Sisal fiber in the matrix after 7 days of curing [130] and (**d**) Jute fiber in the matrix after 28 days of curing [42].

Wongsa et al. [88] conducted a comparative study on GPs without fiber addition and those with sisal fiber, coir fiber, and glass fiber addition at 7 days. The results show that for fiber-reinforced GPs, SEM micrographs show that the surface of sisal fiber and coir fiber is rough and irregular, while the surface of the glass is smooth and uniform. As shown in Figure 6a. It can be shown that the rough surface of natural fiber enhances the bond between fiber and GP composite and improves the tensile and flexural strength of the matrix.

Frydrych et al. [70] showed by scanning electron microscopy that the joint fracture patterns of flax and basalt and fiber-free composites were different. Both flax fibers and basalt fibers exhibit reinforced properties in GPs, which transfer tensile loads during bending. Because of its rough surface, flax fibers are better anchored to GP substrates than basalt fibers with smooth surfaces. Basalt fibers have high tensile strength, but because of their smooth surface, they do not transfer the maximum possible tensile stress. Therefore, the failure mechanism of flax and basalt fiber composites is different. Figure 6a shows the fracture morphology of flax fibers in a GP matrix. This failure mode in such composites corresponds to reference [88]. In the case of basalt fibers, Figure 6b, these fibers do not break but are stripped from the GP. This is due not only to the high tensile strength of basalt fibers but also to their smooth surface.

Figure 6c,d show micrographs of sisal fibers in the 7-day and jute fibers in the 28-day matrix, respectively [130]. It can be seen that after 7 days of curing, the early form of the fibers embedded in the matrix is still intact, with no surface defects caused by mineralization or corrosion. In contrast, after 28 days of hydration, a space was found around the jute fibers due to the drying shrinkage of the cement matrix and fibers. The fibers still retain their original appearance, except for some signs of mineralization.

## 6. Early Shrinkage and Deformation of PFRGs

In contrast to cement-based materials, water does not participate in hydration during polymerization. In this process, the purpose of adding water is to provide a workable mixture. Over time, this excess water will dissipate and cause significant shrinkage of the GPs [131]. Shrinkage causes tensile stress, resulting in internal cracks. The fibers in fiber-reinforced GPs can resist this stress and reduce stress concentration at the crack tip to control its development [106].

PF can reduce the shrinkage of the matrix [132]. Some studies have shown that the main factors controlling the drying shrinkage of fiber-reinforced GPs are fiber type, fiber modulus, fiber content, aspect ratio, and the interaction of fiber binder [133]. The mechanisms for improving toughness include cracking arrest at the fiber-matrix interface and increasing the crack path through the high aspect ratio fiber [134]. Normally, when a crack begins to appear, all the tension at that location is carried by the fiber. A new crack will appear at a different location if the force it can withstand increases without breaking or pulling out the fiber at the crack location. As a result, the fibers in this area will be activated and the force will be transferred. This process will cause multiple cracking until the fibers fail or are pulled out of the matrix.

**Figure 7:**
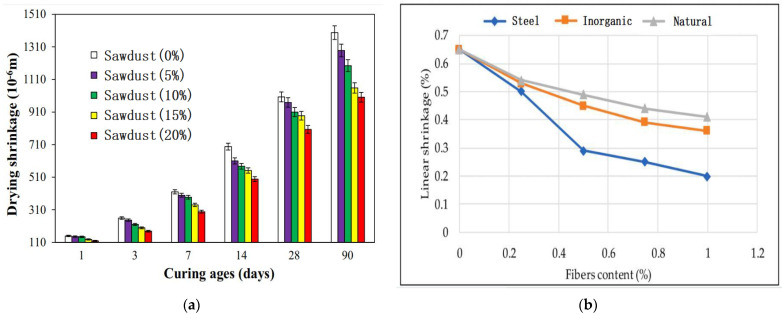
Effects of fibers on shrinkage of GP matrix: (**a**) Effects of Sawdust content and curing time [62]; (**b**) Effects of different fiber content [106].

Although the mechanical properties of GPs containing PFs have been studied extensively, the effects of these fibers on the drying and shrinkage properties have been significantly less reported. Su et al. [106] observed that the addition of PFs to GP slurry reduced shrinkage. However, the shrinkage resistance of the GPs containing PFs is relatively lower than that of the GPs containing steel fibers or inorganic fibers. Figure 6 shows the effect of different PFs on the shrinkage of the GP matrix. Figure 7a shows that the shrinkage increases gradually with the extension of curing time. Figure 7a,b show that the fiber content is inversely proportional to the shrinkage rate.

Fibers can act as bridges in cement-based materials to prevent the spread of cracks. The morphology of the fibers has a great influence on their bonding properties with the cementitious matrix. The main function of fiber is not to improve the compressive strength of composites but to improve its toughness and control the further development of matrix cracks.

Alomayri et al. [102] found that the fracture toughness of GP reinforced with 0.5% cotton fibers was 1.12 MPa higher than that of pure GP. This significant enhancement in fracture toughness is due to fiber pull-out, fiber fracture, and fiber bridging. The addition of 0.7% and 1.0% cotton fiber decreased the workability of the GP matrix. If this problem is overcome by increasing water use, other adverse effects such as increased porosity and microfractures may result. This will result in less binding of the fiber-matrix interface, therefore reducing the stress transferred from the matrix to the fiber.

## 7. The Recent Advances of PFRGs

GPs are cost-effective, environmentally friendly, and consume relatively little energy. However, the early tensile strength of GPs is relatively poor, and the mechanical properties of GPs can be improved by adding PFs.

There are many studies on the mechanical properties of PFRGs, including the durability and degradation of PFs in a matrix. In recent years, more and more types of fibers have been used as reinforcements in a GP matrix. The recent advances of PFRGs are mainly reflected in the following aspects:

Various recycled reinforcements have been used to prepare PFRGs, such as wastepaper sludge, waste cotton stalk, bagasse, and straw particles;

Due to the need for intelligent construction technology, good early performance of PFRGs, including flowability and buildability, is becoming increasingly important, such as the development and preparation of 3d printing PFRGs;

As the admixture of composites, nanomaterials are added to the PFRGs to improve the physical and mechanical properties of the matrix, such as nanoclay and nanocellulose;

A variety of PFs pretreatment methods are used to improve the characteristics of PFs, to enhance the performance of PFRGs.

## 8. Conclusions

The early properties of PFRGs are studied, including the rheological properties of freshly mixed GP slurry, the early compressive strength and flexural strength of GP, and the early shrinkage deformation and cracking of GP.

The addition of PFs greatly delays the setting time of cement-based composites. However, unlike cement, the addition of PFs shortens the setting time of GP due to the heat that may be generated during the degradation of PFs.

The degree of workability loss of different types of fiber-reinforced GP mixtures is affected by fiber type, aspect ratio, and content in the mixture.

PF can improve the early flexural strength of composites and has the function of toughening and strengthening composites. The higher the content of cellulose, the greater the toughening and strengthening function.

Compared with traditional fibers, the influence of PFs on the early performance of GPs mainly has the following problems:

In an alkaline environment, cellulose, hemicellulose, and lignin of PFs are easily hydrolyzed to produce carbohydrate substances. However, saccharides can hinder the solidification of GPs and inhibit the strength development and setting time of cementitious materials during the polymerization, which will have a negative effect on the early mechanical properties of GPs. The characteristics of PF cause its water absorption, variability, and cementitious material bonding to be poor. Because of the high water absorption, the effect of PF on the workability of the GP matrix is higher than that of steel fiber and inorganic fiber.

Through this study, the application of PFs to GPs as reinforcing materials has an adverse effect on the early performance of composites, mainly on the fluidity of mixtures. To solve these problems, PFs can be pretreated with chemical modification to solve them.

## Figures and Tables

**Table 1:** Partial properties of PF reinforcement in related studies.

Fiber Type	Fiber Name	Density/(g·cm^−3^)	Tensile Strength/MPa	Modulus/GPa	Elongation/%	Length/mm	Diameter/mm	Content/%	Ref.
Bast	Flax	1.50	660	39.5	1.2–3.2	-	0.002	4.1 **	[32,33,34,35,36]
	Hemp	1.40–1.50	270–900	23.5–90.0	2.0–4.0	0.5–8.0	-	0.5, 1 *	[37,38,39,40]
	Jute	1.46	393–800	10.0–30.0	1.5–1.8	10.0	0.057–0.093	0.5–10 **	[41,42,43,44]
	kenaf	1.40	152–241	-	3.0–4.4	2.0–4.0	0.076–0.098	0.75–1.5 *	[45]
Leaf	Sisal	1.45	363–700	9.0–38.0	2.0–7.0	3.0	0.500	1 *	[41,46,47,48,49,50]
	Phoenix sp.	1.25	-	-	-	40.0	0.576	1, 2, 3, 4 **	[51]
Seed	Cotton	1.60	400	4.8	7.0–8.0	30.0	0.200–1.000	0.3–8.3 *	[52,53,54,55,56,57,58]
	Coir	1.25–1.50	95–175	4.0–6.0	17.0–51.4	5.0	0.050–0.400	1–5 **	[59,60,61]
Wood	Sawdust	0.79	-	-	-	-	-	4–20 **	[62]
Grass	Raffia palm	-	500	30.0	2.0–4.0	3.0	1.000	1 *	[63,64]
	Bamboo	1.15	518	6.0	1.4	-	0.300–0.380	5 **	[13,65,66]

* indicates the volume content; ** indicates the weight content.

**Table 2:** Different types and contents of fiber-reinforced GP density and water absorption.

Mix	CGM	50SF	75SF	100SF	50CF	75CF	100CF	50GF	75GF	100GF
Dry density/kg/m^3^	1885	1900	1877	1850	1887	1886	1850	1820	1812	1812
Water absorption/%	5.1	5.5	5.5	5.6	5.5	5.4	5.6	4.9	4.9	4.8

SF—Sisal fiber, CF—Coir fiber, GF—Glass fiber, CGM—Controlled GP (without fiber), 100SF—Sisal fiber with 1% volume content.

**Table 3:** Flexural strength test of fiber-reinforced GP composite [MPa].

Fiber	Fiber Content/%	Setting Time/day				Ref.
		7	14	28	90	
Coir	0	5.20	5.00	6.20	-	[59]
	1.00	4.80	5.30	5.30	-	
	2.00	6.10	6.60	6.50	-	
	5.00	4.40	4.30	4.40	-	
Oil palm trunk	0	1.72	2.21	3.45	-	[125]
	0.25	1.89	2.40	3.67	-	
	0.50	1.97	2.57	3.84	-	
	0.75	2.09	2.69	3.97	-	
	1.00	2.23	2.81	4.12	-	
Kenaf	0.75	-	-	5.25	5.46	[45]
	1.00	-	-	5.27	5.80	
	1.25	-	-	6.08	6.40	
	1.50	-	-	5.63	5.90	
Sawdust	0	4.30	5.40	7.70	8.40	[62]
	5.00	4.20	5.40	7.90	8.70	
	10.00	4.80	5.80	8.60	9.40	
	15.00	5.00	7.20	9.40	10.40	
	20.00	5.50	7.50	10.20	11.80	

**Table 4:** Effects of different types and contents of fibers on the strength ratios of GPs.

Strength Ratio	CGM	50SF	75SF	100SF	50CF	75CF	100CF	50GF	75GF	100GF
Flexural to compressive	10.2	15.4	18.5	19.9	16.5	22.1	26.8	10.2	10.8	13.5
Splitting tensile to compressive	6.3	6.3	9.6	10.1	6.3	7.4	8.8	6.7	7.2	8.1

SF—Sisal fiber, CF—Coir fiber, GF—Glass fiber, CGM—Controlled GP (without fiber), 100SF—Sisal fiber with 1% volume content.

## Data Availability

Not applicable.
